# Intestinal microbiota domination under extreme selective pressures characterized by metagenomic read cloud sequencing and assembly

**DOI:** 10.1186/s12859-019-3073-1

**Published:** 2019-12-02

**Authors:** Joyce B. Kang, Benjamin A. Siranosian, Eli L. Moss, Niaz Banaei, Tessa M. Andermann, Ami S. Bhatt

**Affiliations:** 1 Harvard Medical School, Harvard University, Boston, MA 02115 USA; 2 Department of Genetics, Stanford University, Stanford, CA 94305 USA; 3 Department of Medicine, Division of Infectious Diseases and Geographic Medicine, Stanford University, Stanford, CA 94305 USA; 4 Clinical Microbiology Laboratory, Stanford University Medical Center, Stanford, CA 94305 USA; 5 Department of Pathology, Stanford University, Stanford, CA 94305 USA; 6 Department of Genetics, Stanford University, Stanford, CA 94035 USA; 7 Department of Medicine, Division of Hematology, Stanford University, Stanford, CA 94305 USA; 8 Department of Medicine, Division of Infectious Diseases, University of North Carolina, Chapel Hill, NC 27599 USA

**Keywords:** Read cloud sequencing, Metagenomics, Microbiome, Antibiotic resistance, Hematopoietic cell transplantation

## Abstract

**Background:**

Low diversity of the gut microbiome, often progressing to the point of intestinal domination by a single species, has been linked to poor outcomes in patients undergoing hematopoietic cell transplantation (HCT). Our ability to understand how certain organisms attain intestinal domination over others has been restricted in part by current metagenomic sequencing technologies that are typically unable to reconstruct complete genomes for individual organisms present within a sequenced microbial community. We recently developed a metagenomic read cloud sequencing and assembly approach that generates improved draft genomes for individual organisms compared to conventional short-read sequencing and assembly methods. Herein, we applied metagenomic read cloud sequencing to four stool samples collected longitudinally from an HCT patient preceding treatment and over the course of heavy antibiotic exposure.

**Results:**

Characterization of microbiome composition by taxonomic classification of reads reveals that that upon antibiotic exposure, the subject’s gut microbiome experienced a marked decrease in diversity and became dominated by *Escherichia coli*. While diversity is restored at the final time point, this occurs without recovery of the original species and strain-level composition. Draft genomes for individual organisms within each sample were generated using both read cloud and conventional assembly. Read clouds were found to improve the completeness and contiguity of genome assemblies compared to conventional assembly. Moreover, read clouds enabled the placement of antibiotic resistance genes present in multiple copies both within a single draft genome and across multiple organisms. The occurrence of resistance genes associates with the timing of antibiotics administered to the patient, and comparative genomic analysis of the various intestinal *E. coli* strains across time points as well as the bloodstream isolate showed that the subject’s *E. coli* bloodstream infection likely originated from the intestine. The *E. coli* genome from the initial pre-transplant stool sample harbors 46 known antimicrobial resistance genes, while all other species from the pre-transplant sample each contain at most 5 genes, consistent with a model of heavy antibiotic exposure resulting in selective outgrowth of the highly antibiotic-resistant *E. coli*.

**Conclusion:**

This study demonstrates the application and utility of metagenomic read cloud sequencing and assembly to study the underlying strain-level genomic factors influencing gut microbiome dynamics under extreme selective pressures in the clinical context of HCT.

## Background

Metagenomics involves the sequencing of a whole community of microorganisms directly from an environmental sample, such as soil or the human intestinal tract, often without prior knowledge of which species are present within the sample. In silico reconstruction of complete and contiguous genomes for individual organisms within a sequenced population remains a major challenge in the field of metagenomics. This is a challenging problem when using conventional shotgun short-read sequencing and assembly methods because short reads alone may not be able to determine the correct positions of DNA sequences that are both longer than the sequenced DNA fragment length (usually 50–300 base pairs) and present in multiple copies at different locations in the metagenome. The presence of such repeated regions (e.g. insertion sequences or the bacterial 16S rRNA gene) often result in fragmented assemblies where multiple instances of the repeated sequence are collapsed into a single contig instead of correctly placed in between unique flanking regions in multiple genomic locations.

Read cloud sequencing is a relatively new technique that was initially used in the context of human genomics to phase haplotypes [1]. This method has also been termed “linked-read sequencing.” The main difference between read cloud and conventional short-read sequencing is that read cloud sequencing augments the library preparation stage to ultimately generate “read clouds,” which are short-read sequences annotated with long-range information in the form of molecular barcodes. This is achieved by physically partitioning long DNA fragments into nanoliter-scale droplets and subsequently tagging all sequencing reads originating from a long fragment with a droplet-specific molecular barcode. Read cloud sequencing offers a favorable combination of long-range information, high base call accuracy, high throughput, and low input DNA mass requirements [1]. The 10x Genomics Chromium platform is a commercially available read cloud library preparation system that automates the pipetting steps necessary to generate the molecular barcodes. Recently, we developed an approach to adapt read cloud sequencing for metagenomic applications. The resultant barcoded data is deconvolved and genome draft assembly is achieved using a combination of existing standard genome assemblers as well as a custom assembly tool called Athena [2]. We have recently applied the approach to sequence ocean sediment samples and the healthy human microbiome, for which it was able to generate contiguous draft genomes for individual organisms from bacterial mixtures [2].

In this study, we investigate a clinical application of metagenomic read cloud sequencing in the context of hematopoietic cell transplantation (HCT), which is a complex medical procedure used in the treatment of hematologic disorders such as leukemia and lymphoma. During HCT, patients initially undergo intensive treatment with chemotherapy and sometimes radiation therapy; this ‘conditioning regimen’ serves to prepare patients to receive a hematopoietic stem cell graft. Multipotent hematopoietic stem cells derived from bone marrow, peripheral blood, or umbilical cord blood are then infused into the patient to reconstitute all blood cell lines. The procedure can be curative but comes with high risk for complications, including infection and graft-versus-host disease (GVHD), an inflammatory disease where donor immune cells attack the recipient’s healthy tissue. Intestinal microbial dysbiosis preceding and following HCT has been found to be associated with an increased risk for developing bloodstream infections [3]. Previous studies also show that decreased intestinal diversity is associated with development of GVHD and higher overall mortality in HCT [4]. Broad-spectrum antibiotics and other drugs administered during the course of HCT can greatly change the composition of the gut microbiota. In some cases, such microbial dysbiosis leads to domination of the intestine by a few or even a single genus or species, increasing the likelihood of complications like bloodstream infections in these immunocompromised patients [3]. Intestinal domination may happen because certain bacterial strains carry an advantage, such as antibiotic resistance, that enables them to flourish after other antibiotic-sensitive commensal microbes are eliminated. While intestinal domination is relatively common in this patient population, the process by which it occurs is not well-understood.

Herein, we apply the metagenomic read cloud sequencing approach to patient stool samples collected over multiple time points pre- and post-HCT to elucidate microbiome dynamics in response to extreme selective pressures during HCT. We find that antibiotic exposure is associated with intestinal domination by *Escherichia coli* in our study subject. Read cloud sequencing, but not short read sequencing alone, was able to identify many antibiotic resistance genes within the dominating strain of *E. coli*. Thus, we postulate that the gut domination observed was the consequence of enhanced fitness of this organism in the presence of antibiotics.

## Methods

### Sample preparation and sequencing

As part of our original previously published investigation of bloodstream infections in HCT recipients [5], we performed a retrospective cohort study, approved by the Stanford institutional review board under IRB protocol #42053 (principal investigator: A.S.B.). Informed consent for weekly stool sample collection on all Stanford HCT patients was obtained under protocol #8903 (principal investigator: David Miklos). All fresh stool samples were placed at 4 °C immediately upon collection, aliquoted into 2 mL cryovial tubes within 24 h, and stored at − 80 °C.

One study subject undergoing HCT was unique in having a simultaneous *E.coli* and Methicillin-resistant *Staphylococcus aureus* (MRSA) bloodstream infection [5]. Furthermore, this patient also had a total of five longitudinal stool samples (denoted A-E) in addition to the *E. coli* isolate cultured from the bloodstream infection available for sequencing. While MRSA was not found in the patient’s stool sample, the *E.coli* bloodstream isolate appeared indistinguishable from the same strain in the intestine using short-read sequencing [5]. We chose to further investigate this patient’s samples using read cloud sequencing for even more precise longitudinal strain-level analysis.

From the frozen stool samples, we isolated microbial cells from stool debris by differential centrifugation following a previously described protocol [6]. 400 mg of frozen stool was vortexed with 1 mL 0.9% saline solution for 30 s, then centrifuged at 3000 rpm (645 g) for 2 min. The pellet containing stool debris was discarded, and the supernatant was centrifuged at 10,000 rpm (7168 g) for 3 min to spin down bacterial cells. The saline supernatant was discarded, and the differential centrifugation process was repeated with 1 mL of phosphate-buffered saline (pH 7.4) to acquire a purified microbial pellet.

For read cloud sequencing, we extracted high-molecular-weight DNA from the purified microbial pellet using the Gentra Puregene Yeast/Bacteria Kit following the manufacturer’s protocol with the following modifications to increase DNA yield: increased lytic enzyme volume to 5.0 μL and increased protein precipitation solution to 130 μL. For conventional sequencing, we extracted DNA directly from frozen stool using the Qiagen QIAamp DNA Stool Mini Kit modified with an added step after addition of buffer ASL in which the samples underwent seven alternating 30-s cycles of beating with 1 mm diameter zirconia beads in a bead beater (Biospec Products) and chilling on ice. The extracted DNA was visualized by agarose gel electrophoresis, and concentration estimations were performed for both Qiagen and Puregene DNA using Qubit fluorometric quantitation. The concentration of DNA extracted for time point B was too low to be used as input for read cloud sequencing; therefore, the read cloud sample for time point B was excluded from downstream processing. For all other time points, we removed small (< 10 kb) DNA fragments by size selection prior to read cloud library preparation using a BluePippin agarose electrophoresis instrument.

The size-selected high-molecular-weight DNA was used as input for read cloud library preparation. We prepared 10x Chromium libraries using the Chromium instrument and reagents from 10x Genomics (Pleasanton, CA). Additionally, we prepared conventional Illumina Truseq libraries for all five time points (A-E) as well as the bloodstream isolate according to the Illumina Truseq Nano protocol. We quantified library fragment size using a Bioanalyzer 2100 instrument (Agilent Technologies). The four 10x Chromium libraries were multiplexed and sequenced on one lane of Illumina HiSeq 4000 using 2 × 150 bp paired-end reads (11–16 Gb of sequence coverage per library). The Illumina Truseq stool libraries were multiplexed and sequenced on an Illumina HiSeq 4000 instrument using 2 × 101 bp reads (4–5 Gb of sequence coverage per library).

The bloodstream bacterial isolate of *E. coli* was collected and stored by the Stanford Health Care Clinical Microbiology lab, as part of the previously published investigation of bloodstream infections in HCT recipients [5]. We extracted isolate DNA from colonies grown in small volume liquid culture following the manufacturer’s protocol for the Gentra Puregene Yeast/Bacterial Kit and sequenced the Illumina Nextera XT library on an Illumina HiSeq 4000.

### Quality control of reads

The samples were demultiplexed using Illumina’s bcl2fastq v2.19. For the read cloud libraries, we extracted the 16 bp 10x barcode from each read using the Long Ranger Basic pipeline (10x Genomics). Next, we performed identical quality control and filtering procedures for raw reads generated from all stool libraries (both read cloud and conventional): read quality was assessed with FastQC v0.11.4 [7] and quality trimming was performed with cutadapt v1.8.1 using a minimum length of 60 (−m 60), minimum terminal Phred quality cutoff of 30 (−q 30,30), and N-end trimming (−trim-n) [8].

### Taxonomic classification of reads and diversity calculation

To measure the microbial composition of our short-read sequencing samples, we used the Kraken2 taxonomic sequence classifier with default parameters [9] and a comprehensive database containing all bacterial and archaeal genomes in Genbank assembled to “complete genome” or “chromosome” quality as of October 2018. Kraken2 classifies individual reads by mapping all *k*-mers (*k* = 35) to the lowest common ancestor genome in the database. Bracken [10] was then used to estimate species abundance. The Shannon diversity index was calculated for each sample at the species level using the R package Vegan (version 2.5–4) [11]. Shannon diversity was calculated on samples rarefied to 7,360,000 paired-end reads, the number in the lowest covered file.

### Generation of organism draft genomes

We assembled the quality-controlled reads for both the read cloud and conventional libraries using the short-read assembler MEGAHIT v1.1.3 [12], which first builds a succinct de Bruijn graph from *k*-mers, then forms assembled contigs by finding paths through the graph. We performed no further assembly for the conventional samples (the MEGAHIT contigs constituted the final contigs comprising the draft genomes). For read cloud samples, we used BWA v0.7.10 to perform sequence alignment of the raw reads against the MEGAHIT contigs [13]. We then used the Athena assembler to further assemble the MEGAHIT seed contigs. Athena takes as input the barcoded reads (FASTQ), the seed contigs (FASTA), and the alignment file (BAM), and it returns contigs assembled with read clouds (see [2] for full details of Athena).

Next, we clustered the individual contigs generated from Athena into bins representing nearly complete organism genomes. Binning was achieved by using four established metagenomic binning tools: MetaBAT2 [14], MyCC [15], CONCOCT [16], and MaxBin 2.0 [17]. We then used DAS Tool to integrate the results from the various binning methods to yield a single set of non-redundant bins with maximal coverage of single-copy core genes [18]. We assigned a taxonomic classification to each individual contig using Kraken2 [9]. We assigned a taxonomic designation to an entire bin if greater than 60% of contigs in the bin shared the same Kraken2 identification. For each resulting bin, which represents an organism draft genome, we used QUAST to assess the size and contiguity of the assembly [19]. We used CheckM to calculate metrics of genome completeness (existence of expected core genes) and contamination (duplication of core genes expected to exist in single copy) for each draft genome [20]. We used the circlize package in R [21] to visualize and compare the assemblies and Prokka [22] to predict the protein-coding genes in each contig.

### Comparative genomic analysis

To quantify the similarity between the various *E. coli* strains across time points (A, C, and D) and between the stool and bloodstream isolate, we used the NUCmer script within MUMmer v3.23 to perform pairwise alignment of the *E. coli* draft genomes from each pair of samples [23]. We also included the full genome for extraintestinal pathogenic *E. coli* strain S88 (NCBI accession CU928161.2) in the analysis as a comparison. For each pair of assembled draft genomes of the various *E. coli* strains, we calculated the percent nucleotide identity, number of single nucleotide polymorphisms (SNPs), and total number of aligned bases. Additionally, we generated syntenic dotplots for each pairwise comparison using the mummerplot script with layout option (−l), which reorders and orients the contigs to the main diagonal of the plot for optimal viewing [23].

A reference-guided assembly method was used to compare species present at multiple time points when species were too lowly abundant to obtain unbiased bins. For both conventional and read cloud sequencing, reads were aligned against the NCBI reference genome for a given species with BWA [13], mapped reads were extracted with SAMtools [24] and assembled with metaSPAdes [25]. Athena assembly was conducted on read cloud data. Resulting contigs were filtered to a minimum length of 500 bp, and pairs of time points were aligned with MUMmer. Only alignments with > 100 kb 1–1 aligned sequence were reported.

### Antibiotic resistance gene detection

We detected the presence of antibiotic resistance genes within contigs generated from each sample by aligning the predicted protein-coding genes against the Comprehensive Antibiotic Resistance Database (CARD), a curated database of genes known to be determinants of antibiotic resistance [26]. The “protein homolog” model of the CARD database was used in order to minimize false positives. We performed the alignment using DIAMOND [27] and filtered the results to sequences exceeding both 90% identity and 90% coverage of the reference sequence in CARD.

## Results

### Microbiome composition and diversity across the clinical time course

**Fig. 1:**
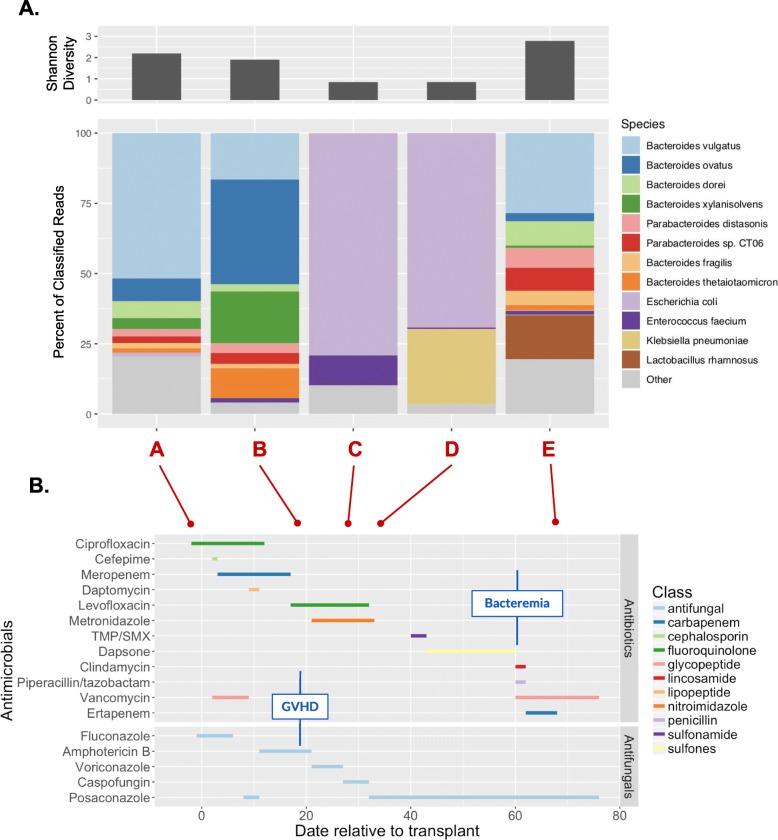
**A** Shannon diversity and composition of the intestinal microbiome of the study subject across five time points over the course of HCT obtained from species-level taxonomic classification of conventional short-read samples. Each bar represents one stool sample, where colors represent different species and thickness indicates relative readcount attributed to that species within the sample (proportion of total reads classified to the species level). “Other” represents species comprising < 2% readcount. Microbial diversity decreases to a period of domination by *E. coli* (time points C and D) followed by recovery of diversity (time point E). **B** Clinical time course of the study subject. The *x-*axis denotes number of days after transplantation. Dates on which a stool sample was collected are marked by red dots. Each row portrays the start and end date of administration of an antibiotic (antibiotic class indicated by the color of the line). The timing of GVHD onset and bloodstream infection (bacteremia) are marked

Stool samples were collected from the patient over five time points spanning 70 days. The samples (denoted A-E) correspond to days − 2, + 19, + 27, + 33, and + 68 relative to transplantation. Figure 1 plots the microbial diversity as measured by the Shannon diversity index as well as the species-level taxonomic composition (from metagenomic classification of conventional short-read data) of the patient’s gut microbiome over time in relation to when the patient was administered various antibiotics. Across the time course spanning 70 days, Shannon diversity was found to decrease markedly from time point A through a period of intestinal *E. coli* domination (samples C and D) before completely recovering by time point E. The patient exhibits the *E. coli* gut domination after the time of GVHD onset on day + 19 and before the clinical manifestation of the *E. coli* bloodstream infection on day + 60.

**Fig. 2:**
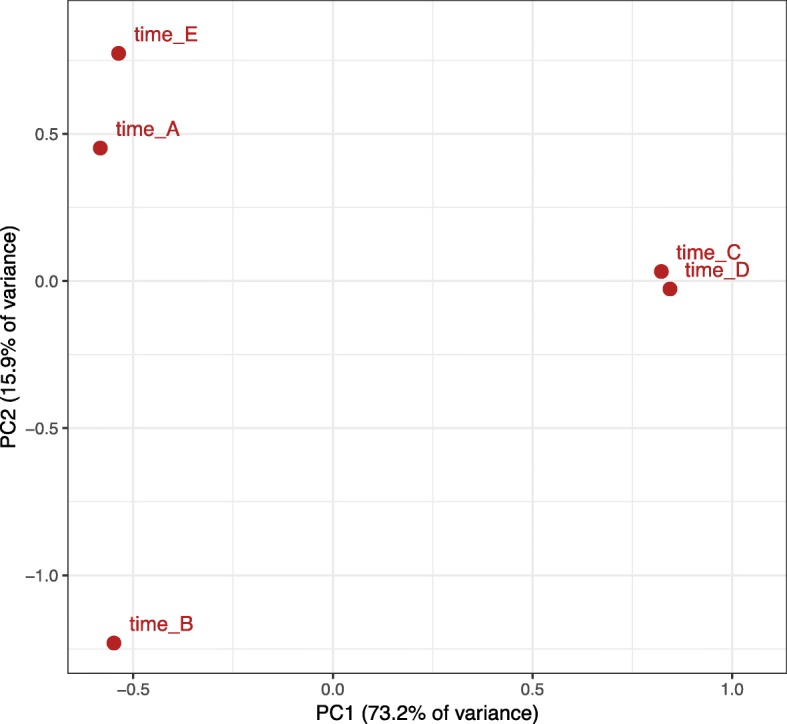
Principal Coordinate Analysis (PCoA) of microbiome content classified at the species level (Bray-Curtis beta diversity metric). Most of the variation is captured in the *x*-axis and separates *E. coli* dominated samples from the rest. Time points A and E are closer together than time point B, showing the recovery of a similar microbiome community following transplant

We calculated the Bray-Curtis dissimilarity index between pairs of samples and performed Principal Coordinates Analysis (PCoA) to visualize microbiome composition (Fig. 2). Most of the variance in the PCoA plot is captured by the stark difference in *E. coli-*dominated samples (C and D), as expected. Time points A and E are more similar than time points A and B, suggesting recovery of a similar microbial community. However, we note that time point B occurred after the completion of the transplant and engraftment process, while the patient was exposed to several antibiotic agents. Sample E also has significant representation of species not found in time point A, including a 16% fraction of *Lactobacillus rhamnosus.*

Recovery of diversity and original microbial community structure after HCT could occur through persistence of microbes in very low fractions, acquisition of new microbes following the HCT process, or a combination of both. To evaluate these options, we examined if microbial genomes assembled from identical organisms at multiple timepoints had high nucleotide similarity (see Methods). Of species present at a relative abundance greater than 2% in multiple samples, 8 species are present at time points A, B and E. Five out of 8 species had > 99.9% nucleotide similarity between time points A and B, likely indicating the same dominant strain is present at both time points. Lower A-B similarity for other species could be the result of different strain populations between time points or poor assembly, as these species had < 1 Mb of assembled and aligned sequence.

In all cases, sequences assembled from species present at time points A and E had < 99.5% similarity (Additional file 1). Interestingly, *Enterococcus faecium* is > 99.9% similar between samples B, C and D, but much different at time point E (~ 96% similarity, E compared to other time points). This suggests the same dominant strain of *Enterococcus faecium* is retained though the *E. coli* domination event, but a different strain is acquired or dominant by time point E. Similar results were achieved with short-read and Athena assemblies, when data were available. Taken together, these results suggest that dominant original strains are not retained in the microbiome through the clinical time course. However, this analysis cannot rule out lowly abundant strains that did not contribute to the genome assembly, which could be present either before or after the *E. coli* domination event.

### Assembly of draft genomes

We separately performed both conventional short-read assembly (MEGAHIT) and read cloud assembly (Athena) and binned the resulting contigs into draft genomes for individual organisms present within each metagenomic sample (see Methods). We assessed the draft genome bins using CheckM and defined “high-quality” bins as attaining > 90% completeness and < 5% contamination, following a previously described standard [28]. By this standard, read cloud sequencing and Athena assembly produced 16 high-quality draft genomes for time point A (listed in Table 1), whereas conventional short-read sequencing and assembly produced 6 high-quality genomes. Binning results and assembly metrics for Athena draft genomes generated for each time point can be found in Additional file 2.

**Table 1:** Athena draft genome assemblies generated for sample A

Organism	Size (Mb)	Coverage	Completeness	Contamination	N50
*Catenibacterium sp.*	2.57	50.16	100	0	160,908
*Erysipelotrichaceae bacterium*	4.33	50.59	100	3.77	498,545
*Streptococcus thermophilus*	1.74	21.49	99.89	0.58	49,696
*Faecalibacterium prausnitzii*	2.95	45.28	99.66	3.17	292,610
*Eubacterium rectale*	3.32	178.86	99.52	0.72	375,749
*Flavonifractor plautii*	3.6	52.02	99.33	0.81	983,109
*Eubacterium (Genus)*	2.91	29.92	99.33	2.68	148,852
*Bacteroides vulgatus*	5.35	629.82	98.5	0.19	502,539
*Escherichia coli*	4.96	20.48	98.4	0.58	70,983
*Parabacteroides distasonis*	5.28	77.48	98.27	0.83	455,277
*Streptococcus parasanguinis*	2.1	17.92	97.89	0	46,401
*Clostridium sp.*	3.08	18.87	97.63	0	42,920
*Bifidobacterium longum*	2.47	44.91	97.62	1.08	111,224
*Blautia sp.*	3.09	34.29	96.2	0	272,530
*Bacteroides ovatus*	5.98	56.70	94.61	1.87	529,675
*Blautia sp.*	3.16	26.35	92.83	2.22	140,920

**Fig. 3:**
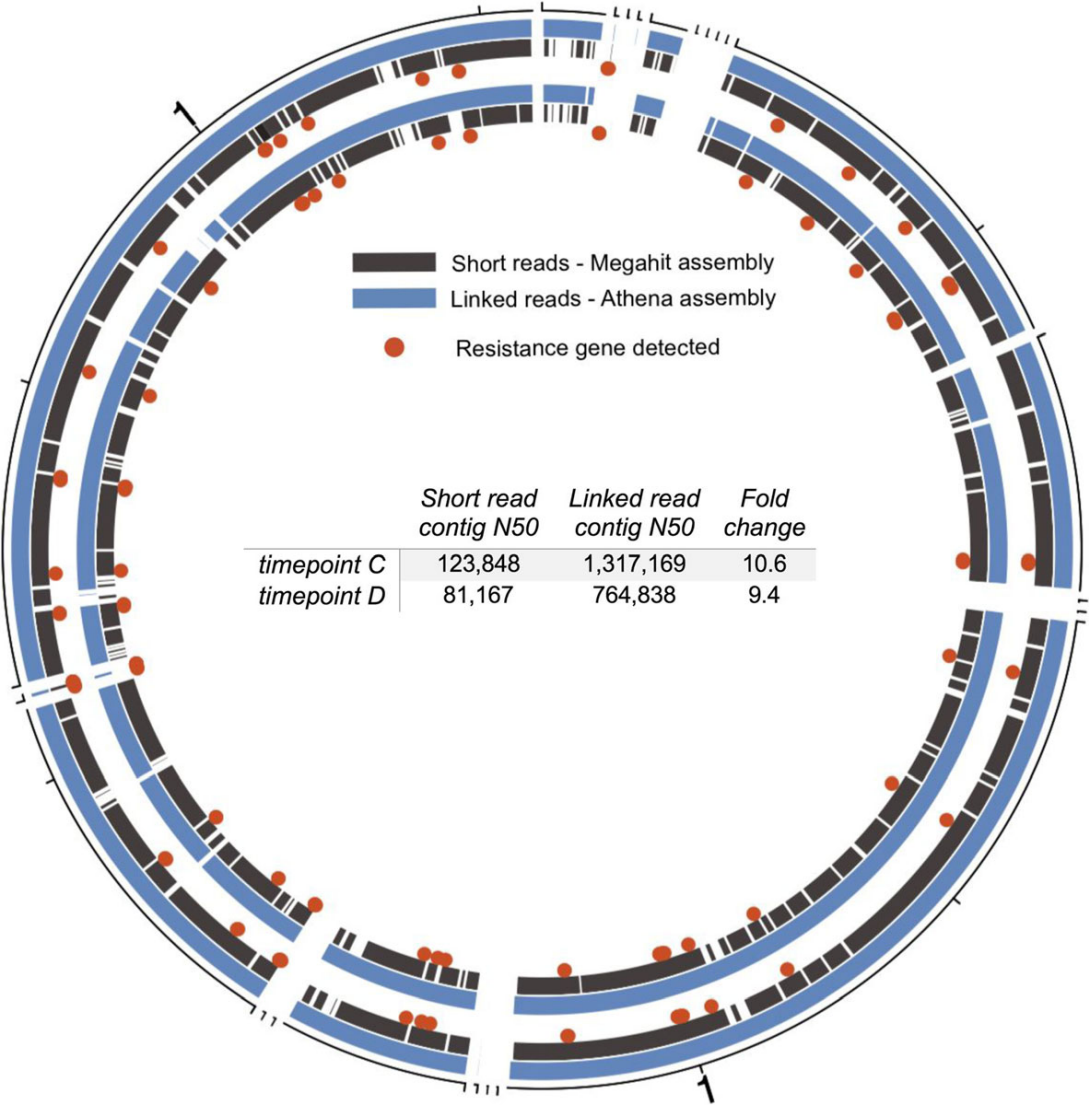
Circos plot showing *E. coli* draft genomes for sample C (outer track) and D (inner track) constructed with read clouds and Athena assembly (blue) compared to conventional short reads and MEGAHIT assembly (dark grey). Athena assembly demonstrates enhanced contiguity with an approximately 10-fold improvement in N50 for both samples compared to the conventional assembly. Red dots mark genomic locations where resistance genes were detected. Red dots located at breaks in the grey track identify resistance genes detected in the Athena assembly but were missing from at least one of the short-read assemblies

Figure 3 shows a visual comparison of the *E. coli* draft genomes generated using read clouds compared to conventional sequencing for time points C and D, when *E. coli* comprises the most abundant organism in the sample. Compared to the conventional assembly, the Athena assembly demonstrated an order of magnitude increase in contig N50. An assembly’s N50 is a metric of contiguity defined as the length of the shortest sequence such that 50% of the entire assembled genome is included in contigs of greater or equal length (higher N50 indicates greater contiguity). The draft genome for sample C was the most contiguous and complete *E. coli* assembly, containing 5.16 Mb of sequence in 23 contigs with an N50 of 1.32 Mb. Overall, these results support our previous finding [2] that read cloud sequencing and Athena assembly improves the reconstruction of genomes of individual organisms within microbial mixtures.

### Detection of resistance genes

We aligned the predicted protein-coding sequences from the Athena-assembled metagenomes for samples A, C, D, and E against the Comprehensive Antibiotic Resistance Database (CARD) database, which yielded 87 (71 unique), 72 (72 unique), 101 (86 unique) and 15 (11 unique) resistance genes, respectively. Herein, we use the term *resistance gene* to refer to any gene present within the CARD database, which comprises genes known to confer antibiotic resistance and regulators of such genes. In the entire metagenome assembled for sample A, we detected several resistance genes present in multiple copies: *tetO* (7 copies), *cfxA3* (5 copies), *mefA* (3 copies), *tetQ* (3 copies), *tet(40)* (2 copies), and *ermF* (2 copies). We found that copies of identical resistance genes occurred both within the genome of the same organism and among different organisms. For instance, *tetO* was present on 3 contigs that all belonged to the *Lachnospiraceae* bin, and it was also present in single copy in draft genomes classified as *Blautia sp., Clostridium*, *Eubacterium rectale*, and *Ruminococcus gnavus*. Inspection of the genomic regions of the 3 *Lachnospiraceae* contigs containing *tetO* revealed that the regions with the resistance gene share some homology but are not completely identical. Note that no resistance gene duplication was observed for sample C. For sample D, a set of 13 resistance genes (*acrB, acrD, baeR*, *cpxA*, *CRP*, *emrB*, *emrR*, *marA*, *mdtB, mdtC, msbA, patA*, and *sul1*) was detected in the draft genomes of both *E. coli* and *K. pneumoniae*. Although both organisms share this same set of genes, we did not find evidence for horizontal gene transfer because the genes themselves are not identical (different numbers of mismatch from the reference), and the contigs on which the genes are present have homology in the region of the resistance genes but are not completely identical as determined by alignment dotplots of the contig pairs. For sample E, the *dfrF* gene appeared in 5 distinct copies in 4 different organism bins. Positive selection for the *dfrF* gene may have potentially occurred given that trimethoprim was administered to the patient prior to time point E.

Performing the equivalent resistance gene analysis on the conventional sequencing data for samples A, C, D and E revealed 27 (27 unique), 84 (84 unique), 94 (82 unique) and 9 (9 unique) resistance genes, respectively. Compared to read cloud assembly, a greater proportion of resistance genes detected in the conventional data are unique (in single copy) within their assembly as genes present in multiple copies are collapsed into a single sequence in the absence of barcode information. The specific resistance genes detected within each read cloud and conventional sample as well as alignment metrics are listed in Additional file 3. These results show that the ability to resolve numerous copies of the same resistance gene present in one or multiple distinct organisms within the proper genomic context is a notable technological advantage of the read cloud sequencing over conventional methods.

### Comparative genomic analysis of *E. coli* strains

We postulated that comparison of the *E. coli* draft genomes across time points would reveal genomic differences between the *E. coli* assemblies. Assuming that the assembled *E. coli* genome for a given time point represents the most abundant strain of *E. coli* in the sample, significant genomic differences across time could indicate acquisition of a new strain, selection and subsequent outgrowth of a previously low-abundance strain, or possible remodeling of the genome. We also hypothesized that the particular strain of *E. coli* producing the bloodstream infection could be traced back to the gut microbiome based on our previous findings in [5]. To assess *E. coli* strain similarities, we aligned pairs of *E. coli* draft genomes from the various stool time points and the bloodstream isolate against each other (see Methods). Table 2 lists the average percent nucleotide identity, total number of SNPs, and total bases aligned for each pair of genomes. We also included NCBI *E. coli* S88 reference genome in the analysis to serve as a comparison to a strain that is also a known extraintestinal pathogen but unrelated to our patient.

**Table 2:** Comparison of *E. coli* strain similarities across time and spatial location

Draft genome 1	Draft genome 2	Total bases aligned	Average percent identity	Total number SNPs
Assembly A	Assembly C	4,965,009	99.98	371
Assembly C	Assembly D	5,050,613	99.91	3811
Bloodstream isolate	Assembly C	5,056,888	99.99	182
Bloodstream isolate	Assembly D	5,002,210	99.91	3742
*E. coli strain S88 (NCBI)*	Assembly C	4,410,742	98.61	56,513

We discovered that the dominant intestinal *E. coli* strains present in samples A, C, and D contain relatively few SNPs and share extremely high nucleotide identity. The number of SNPs ranged from 371 to 3811 (compared to 56,513 SNPs with the S88 reference) and percent nucleotide identity ranged from 99.91 to 99.98% (compared to 98.61% identity with the S88 reference). Somewhat interestingly, the bloodstream isolate (day + 60) genome most closely matched the draft genome from sample C (day + 27) with 182 SNPs and 99.99% nucleotide identity, even though the patient’s clinical manifestation of bloodstream infection occurred after time point D (day + 33) with 3742 SNPs and 99.91% identity. The low number of SNPs and high percent identity between the stool sample *E. coli* strains and the bloodstream isolate reveal that the same *E. coli* strain existing in the patient’s intestine prior to HCT likely persisted in spite of antibiotics, expanded to dominate the gut, and also eventually caused the patient’s bloodstream infection. Our group initially analyzed the short-read libraries of these samples via an orthogonal bioinformatic approach as described in [5], which also suggested that the intestine was the source of the bloodstream strain for this patient.

**Fig. 4:**
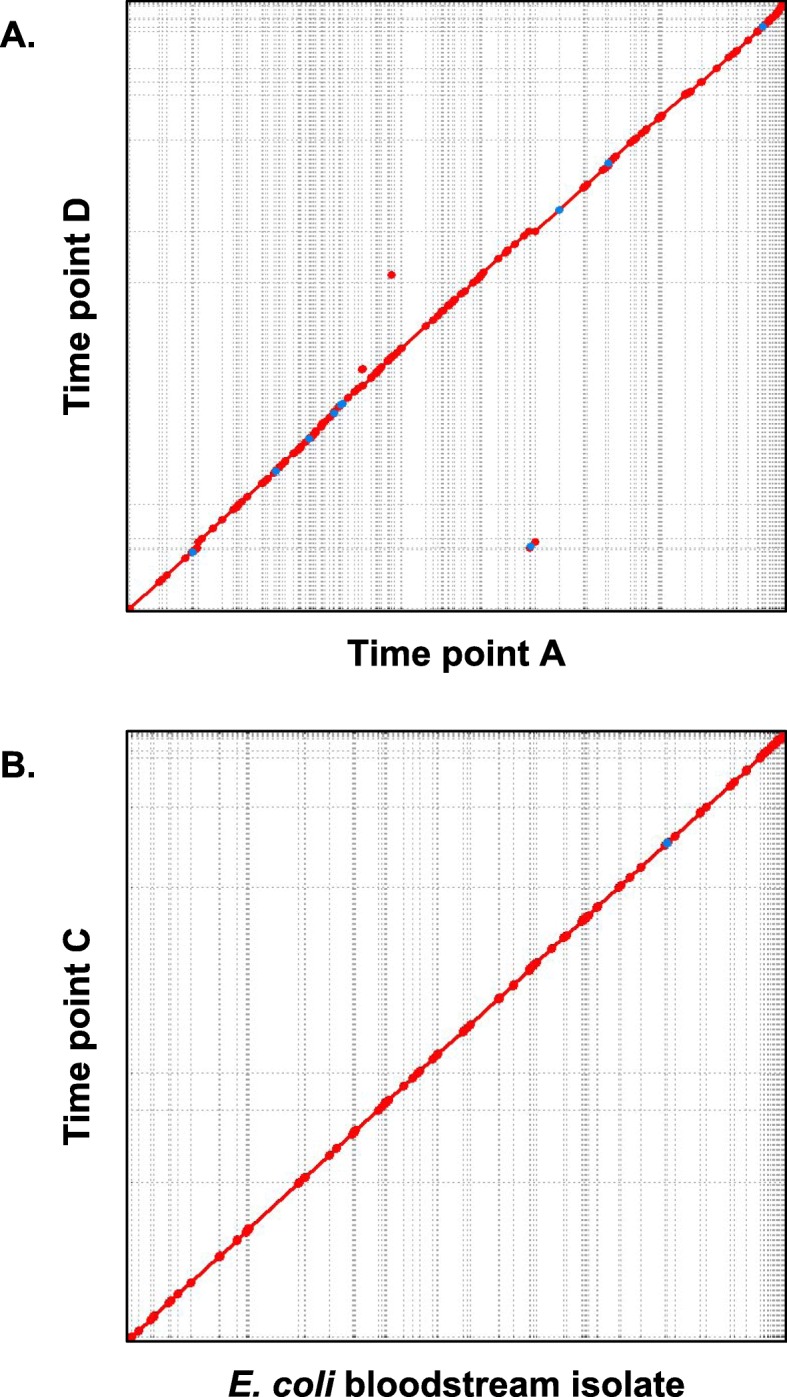
Syntenic dotplots comparing *E. coli* strains across time points and between the intestine and the bloodstream. Regions of sequence identity are marked by colored lines. **A** Sample A draft genome (*x*-axis) compared to sample D draft genome (*y*-axis). **B** Bloodstream isolate genome (*x*-axis) compared to sample C draft genome (*y*-axis). The near-perfect correspondence reveals that the bloodstream isolate is concordant with and thus likely originated from the intestinal microbiome

In order to ascertain whether any large-scale genomic island incorporation or genomic remodeling took place in the dominant *E. coli* strain over time, we visualized pairwise genome alignments of the various strains as syntenic dotplots, which can compare two genomes to each other. Each main axis represents the entire length of one genome being compared, and a colored dot is plotted at regions where the genomic sequences match between the two genomes (areas of synteny). For example, comparing two completely identical genomes would produce a dotplot with a perfectly contiguous diagonal stretching from the bottom-left to top-right corners. Figure 4 shows the synteny dotplots comparing *E. coli* strains from sample A to sample D and comparing the bloodstream isolate to sample C. Visual inspection of the plots showed no evidence for any large genomic island incorporations. The lack of major discontinuities or inversions provide additional evidence that the strains are genetically equivalent from a genome structure perspective across the various time points and between the gut and the bloodstream.

### Antibiotic resistance genes in pre-transplant *E. coli* strain

Given that the *E. coli* strain dominating the intestine likely originated from a single original strain that persisted through the extreme selective pressures of antibiotic administration, we hypothesized that the pre-transplant (time point A) strain harbored antibiotic resistance genes that potentially aided its survival. By aligning the predicted protein-coding regions of the Athena-assembled *E. coli* draft genome from sample A against the CARD database, we detected 46 known antibiotic resistance genes (Table 3). Functional annotations of these genes revealed that the majority of genes code for proteins related to drug efflux pumps, and others encode known resistance mechanisms to aminoglycosides, bacitracin, and polymyxin. There was also a gene (*CTX-M-27*) that confers extended-spectrum beta-lactamase resistance.

**Table 3:** Antibiotic resistance genes present in pre-transplant *E. coli* genome

Category	Resistance Gene(s)
Beta-lactam resistance	*CTX-M-27*
Aminoglycoside resistance	*kdpE*
Polymyxin resistance	*arnA, pmrC, pmrE, pmrF*
Bacitracin resistance	*bacA*
Efflux pump complex or subunit	*acrA, acrB, acrD, acrE, acrF, emrA, emrB, emrD, emrE, emrK, emrY, marA, mdfA, mdtA, mdtC, mdtE, mdtF, mdtG, mdtH, mdtM, mdtN, mdtO, mdtP, msbA, msrB, patA, TolC, YojI*
Protein modulating antibiotic efflux	*acrS, baeR, baeS, cpxA, CRP, emrR, evgA, evgS, gadW, gadX, H-NS*

Next, we evaluated the fitness of the pre-transplant *E. coli* strain to other organisms present in the same stool sample at time point A by comparing the resistance gene content of *E. coli* to that of the other organisms. Out of the total 87 resistance genes detected in the entire metagenome for sample A, 46 were localized to contigs in the *E. coli* draft genome bin. The remaining 41 genes were distributed widely across many other organisms, with no individual bin containing greater than 5 resistance genes. The organisms containing the second-highest number of resistance genes (each with 5 genes) were classified at the genus level as *Lachnospiraceae* and *Eubacterium*. Because all organisms with a near-complete draft genome possessed no more than 5 resistance genes, our results support a model in which the particular *E. coli* strain present in the subject’s microbiome prior to transplant was able to achieve gut domination over other organisms due to the selective pressures applied by antibiotics.

## Discussion

Our results show that the metagenomic read cloud sequencing methodology allows for more comprehensive and contiguous recovery of individual bacterial genomes from a sequenced community within the gut microbiome of an HCT patient. The improved assemblies allow for augmented detection of antibiotic resistance genes that are present in multiple copies in the metagenome and facilitates comparative genomic analysis to ascertain strain similarity.

Recovery of microbial diversity is expected following HCT, but previous research has shown that the post-HCT microbiome is often different than the pre-HCT microbiome [29]. Our results corroborate these findings as microbiome diversity is restored at time point E without the recovery of the original species and strain-level composition. We find that the assembled genomes for organisms present at time point E compared to other time points are actually quite different (< 99.5 similarity for strains of the same species). Several potential mechanisms could explain this finding: for example, a new strain (either externally acquired or a previously rare strain) may become dominant due to selective fitness advantage; alternatively, drug exposure occurring over the clinical time course may drive widescale mutagenesis of the dominant strain within these organisms.

*Bacteroides* was the most abundant genus in the subject’s microbiome prior to transplantation (sample A). The patient was then administered multiple antibiotics, and the microbiome concurrently developed markedly decreased diversity until becoming dominated by *E. coli*. Previous studies have established *Bacteroides* to be an abundant and prevalent genus in the healthy human gut microbiome; conversely, healthy populations rarely exhibit gut domination by Proteobacteria like *E. coli* [30]. By characterizing the presence of antibiotic resistance genes in the gut metagenome, we discovered that the *E. coli* strain present at time point A, before transplant and before any antibiotic administration, already contained a vast arsenal of antibiotic resistance genes. Increased fitness due to a greater number of resistance mechanisms may have afforded this particular *E. coli* strain a selective advantage, enabling it to survive as other organisms were eliminated by the antibiotics.

In the setting of the specific antibiotics administered to the patient, the survival of the dominating *E. coli* strain may be explained in part by the resistance genes detected in its genome. Preceding the *E. coli* domination observed starting at time point C (day + 27), the patient had received the following antibiotics in chronological order: ciprofloxacin (day − 2 to + 12), cefepime (day + 2 to 3), vancomycin (day + 2 to 9), meropenem (day + 3 to 17), daptomycin (day + 9 to 11), levofloxacin (day + 17 to 32), and metronidazole (day + 21 to 33). The strain’s observed resistance to ciprofloxacin and levofloxacin (members of the fluoroquinolone class of antibiotics) can potentially be explained by multidrug efflux complexes AcrAB-TolC, AcrEF-TolC, EmrAB-TolC, and MdtEF-TolC as well as multidrug resistance proteins MdtH and MdtM, which are all annotated in CARD as potentially conferring fluoroquinolone resistance. The observed resistance to Piperacillin/tazobactam (a penicillin) and cefepime (a cephalosporin) may be attributed to *CTX-M-27*. The patient’s bloodstream infection was due to a highly resistant extended-spectrum beta-lactamase (ESBL) *E. coli* bacteria, and most of the ESBL *E. coli* infections in the U.S. are accounted for by CTX-M-type enzymes [31]. Our analysis did not identify resistance genes that can explain the ability for this particular strain of *E.coli* to survive despite the use of meropenem; however, a decrease in uptake of antibiotics due to a deficiency of porin expression or biofilm formation may possibly be involved [32]. *E. coli* possesses native resistance to daptomycin and vancomycin, which both target Gram-positive organisms.

While this analysis follows a single HCT patient, our findings have broader clinical implications. We demonstrate that the intestinal microbiome of patients can act as a reservoir of antibiotic resistance genes, which may govern which organisms are most predisposed to endure and dominate the gut under the extreme selective pressure applied by antibiotics. Although broad-spectrum antibiotics remain a vital part of our medical armamentarium, the issue of increasing antibiotic resistance strongly argues for their conscientious use. Antibiotics can both select for antibiotic resistance and contribute to the loss of commensal organisms and resulting expansion of a few organisms or even a single organism to the point of gut domination. Further studies are warranted to investigate whether our findings generalize to other HCT patients as well. It is conceivable that the antibiotic resistance gene potential of organisms present prior to transplantation can be used to predict or explain eventual gut domination events or bloodstream infections. Additionally, it is important to note that the resistance genes detected in this study are limited to known antibiotic resistance mechanisms present within the CARD database, and commensals likely have mechanisms of resistance that remain unknown.

## Conclusion

This case study serves as an example of how advanced DNA sequencing technologies can help to illuminate complex biological phenomena occurring within real patients. We explore a clinical application of our recently developed metagenomic read cloud sequencing and assembly approach to study gut microbiome dynamics under the intense selective pressures caused by heavy antibiotic administration in the context of HCT. Because intestinal domination has been linked to poor outcomes in this patient population, we applied read cloud sequencing to longitudinal stool samples of an HCT patient who developed *E. coli* gut domination and a subsequent bloodstream infection. Read cloud sequencing and the Athena assembler provided a higher-resolution characterization of microbiome dynamics surrounding the period of domination than conventional short-read sequencing alone, as it generated draft genomes for constituent organisms in the patient’s microbiome with greater completeness and contiguity. Moreover, the improved assembly using read cloud sequencing enhanced our ability to assemble multiple copies of conserved and repeated sequences (e.g. antibiotic resistance genes) within their proper genomic context.

The generation of high-quality assemblies enabled the genomic comparison of organisms over time. We find that although microbial diversity recovers in our subject post-HCT, for most organisms the original dominant strains are not retained throughout the clinical time course. By performing comparative genomic analysis on the *E. coli* strains between the gut microbiome across time and the bloodstream, we found that a single highly resistant strain of *E. coli* originally residing within the patient’s baseline microbiome prior to HCT and antibiotic treatment persisted to eventually dominate the subject’s microbiome and also instigate the bloodstream infection. By detecting known antibiotic resistance genes within the assembled genomes, we discovered that the *E. coli* strain present before transplant was armed with a large collection of resistance genes whereas other organisms initially present in the same intestinal community lacked such extensive resistance potential. These findings are aligned with a model in which the eventual gut domination by *E. coli* can be attributed to its increased fitness compared to other organisms, leading to its outgrowth under extreme selective pressures. A more comprehensive understanding of microbiome dynamics occurring in HCT could potentially lead to the development of personalized antibiotic regimens based on the gene content of microbial strains within an individual’s microbiome or microbiome-related treatments to improve patient outcomes by preserving or enhancing microbiota diversity during the course of HCT.

## Supplementary information

**Additional file 1.** Pairwise assembly comparison of species across time points. Pairwise genomic comparisons of species present at > 2% in samples at multiple time points. Table lists nucleotide similarity (percent identity) and number of bases aligned, for both read cloud and short-read assemblies.

**Additional file 2.** Draft genomes generated by read cloud assembly. Binning results and assembly metrics for Athena draft genomes generated for all stool samples sequenced using read clouds (separate tabs for time points A, C, D, and E).

**Additional file 3.** Antibiotic resistance genes detected. Resistance genes detected within read cloud and conventional samples by aligning assembled sequences against the CARD database (separate tabs for time points A, C, D, and E, for both read cloud and conventional methods).

## Data Availability

Quality control processed sequencing data with human reads removed can be found for both short read and 10x under the NCBI accession number PRJNA523592.

## Abbreviations

CARD: Comprehensive Antibiotic Resistance Database
GVHD: Graft-versus-host disease
HCT: Hematopoietic cell transplantation
