# Supplementary material for: Intestinal microbiota domination under extreme selective pressures characterized by metagenomic read cloud sequencing and assembly
Source: BMC Bioinformatics. 2019 Dec 2;20(Suppl 16):585. doi: 10.1186/s12859-019-3073-1 (PMC6886166; doi:10.1186/s12859-019-3073-1)
Supplement: Supplementary file 1 — Additional file 1. Pairwise assembly comparison of species across time points. Pairwise genomic comparisons of species present at > 2% in samples at multiple time points. Table lists nucleotide similarity (percent identity) and number of bases aligned, for both read cloud and short-read assemblies. [file 12859_2019_3073_MOESM1_ESM.docx]

**Supplementary Table 1: Pairwise assembly comparison of species across time points.**

|  |  |  | **Short Read Sequencing** | | **Recad Cloud Sequencing** | |
| --- | --- | --- | --- | --- | --- | --- |
| **Species** | **Sample 1** | **Sample 2** | **% identity** | **1-1 alignment length (Mb)** | **% identity** | **1-1 alignment length (Mb)** |
| *Bacteroides dorei* | A | B | 99.96 | 3.62 | *NA* | |
| *Bacteroides dorei* | A | E | 99.40 | 3.32 | 99.38 | 3.35 |
| *Bacteroides dorei* | B | E | 99.40 | 3.32 | *NA* | |
| *Bacteroides fragilis* | A | B | 99.39 | 0.19 | *NA* | |
| *Bacteroides fragilis* | A | E | 94.60 | 0.15 | 94.01 | 0.14 |
| *Bacteroides fragilis* | B | E | 94.31 | 0.17 | *NA* | |
| *Bacteroides ovatus* | A | B | 99.99 | 4.39 | *NA* | |
| *Bacteroides ovatus* | A | E | 97.72 | 3.64 | 97.68 | 3.19 |
| *Bacteroides ovatus* | B | E | 97.71 | 3.75 | *NA* | |
| *Bacteroides thetaiotaomicron* | A | B | 98.57 | 0.55 | *NA* | |
| *Bacteroides thetaiotaomicron* | A | E | 96.95 | 0.24 | 96.83 | 0.17 |
| *Bacteroides thetaiotaomicron* | B | E | 95.99 | 0.27 | *NA* | |
| *Bacteroides vulgatus* | A | B | 99.98 | 4.14 | *NA* | |
| *Bacteroides vulgatus* | A | E | 99.43 | 3.63 | 99.42 | 3.75 |
| *Bacteroides vulgatus* | B | E | 99.42 | 3.64 | *NA* | |
| *Bacteroides xylanisolvens* | A | B | 99.97 | 3.74 | *NA* | |
| *Bacteroides xylanisolvens* | A | E | 97.33 | 2.79 | 97.21 | 2.31 |
| *Bacteroides xylanisolvens* | B | E | 97.33 | 2.84 | *NA* | |
| *Escherichia coli* | A | C | 99.93 | 0.66 | 99.98 | 3.02 |
| *Escherichia coli* | A | D | 99.93 | 0.67 | 99.97 | 3.03 |
| *Escherichia coli* | C | D | 100.00 | 3.93 | 99.99 | 3.97 |
| *Parabacteroides CT06* | A | B | 99.39 | 0.19 | *NA* | |
| *Parabacteroides CT06* | A | E | 98.48 | 3.62 | 98.45 | 3.61 |
| *Parabacteroides CT06* | B | E | 98.48 | 3.63 | *NA* | |
| *Parabacteroides distasonis* | A | B | 99.99 | 3.83 | *NA* | |
| *Parabacteroides distasonis* | A | E | 98.64 | 3.52 | 98.59 | 3.52 |
| *Parabacteroides distasonis* | B | E | 98.64 | 3.52 | *NA* | |
| *Enterococcus faecium* | B | C | 99.97 | 2.54 | *NA* | |
| *Enterococcus faecium* | B | D | 99.92 | 1.88 | *NA* | |
| *Enterococcus faecium* | B | E | 95.85 | 0.92 | *NA* | |
| *Enterococcus faecium* | C | D | 99.92 | 1.88 | 99.99 | 2.73 |
| *Enterococcus faecium* | C | E | 95.85 | 0.92 | 95.71 | 0.88 |
| *Enterococcus faecium* | D | E | 95.90 | 0.69 | 95.7 | 0.88 |
